# Understanding Psychiatrists' Knowledge of Eating Disorders

**DOI:** 10.1192/bjo.2024.289

**Published:** 2024-08-01

**Authors:** Daniel De Silva, Isabella Jurewicz

**Affiliations:** 1 Cwm Taf Morgannwg University Health Board, Llantrisant, United Kingdom; 2 Cardiff and Vale Health Board, Cardiff, United Kingdom

## Abstract

**Aims:**

Wales is the only member of the home nations without Specialist Eating Disorders Training (SEDT) and does not currently have any Specialist Eating Disorder Units (SEDU). This has resulted in varied exposure and experience to eating disorder (ED) psychiatry within psychiatrists working in Wales. Patients with ED have the highest mortality out of all conditions in psychiatry and with hospital admissions for ED on the rise it is important that we understand current attitudes towards ED and use this data to improve understanding and services provided for these patients.

The aims of this project are to

1. Gauge the experience and knowledge of eating disorders of psychiatrists in Wales.
2. Examine attitudes towards management of different risks and which individuals should manage what aspects of care regarding eating disorders.
3. Identify future avenues of development for eating disorders services in Wales.

**Methods:**

An online survey was sent to the 518 members in the Royal College of Psychiatrists in Wales mailing list identifying their current position within psychiatry, experience in working with ED patients and then different questions regarding their attitudes towards ED, ED management, their opinions on development of SEDT posts and their opinions on the development of SEDU in Wales.

There were 36 anonymous responses from doctors working in or around psychiatry in Wales. Responses were collected between March and April 2022 with the survey taking on average less than 5 minutes to complete.

**Results:**

36 individuals answered the survey with consultant/SAS (Senior) level doctors making up 69% of responses, the remaining 31% being psychiatry or GP trainees. Senior doctors mostly comprised general adult or CAHMS specialists, but other specialities were also present. 75% of responses reported some expertise in ED. 50% reported they were confident in the management of ED however there were varying responses when asked about the management of physical health in ED. 89% of responses indicated they would like to see the development of ED psychiatry posts and 78% of responses would like to see the development of SEDU in Wales for severely ill patients.

**Conclusion:**

Many of the responses indicated some exposure to ED however 50% of responders did not feel confident in the management of ED. The development of ED psychiatry posts and SEDU would likely aid in increasing confidence of ED management.

